# Self-care practices regarding diabetes among diabetic patients in West Ethiopia

**DOI:** 10.1186/s13104-019-4258-4

**Published:** 2019-04-08

**Authors:** Mohammed Gebre Dedefo, Balisa Mosisa Ejeta, Getu Bayisa Wakjira, Ginenus Fekadu Mekonen, Busha Gamachu Labata

**Affiliations:** 1 Clinical Pharmacy Unit, Department of Pharmacy, College of Health Sciences, Wollega University, Nekemte, Oromia Ethiopia; 2 Pharmaceutics Unit, Department of Pharmacy, College of Health Sciences, Wollega University, Nekemte, Oromia Ethiopia

**Keywords:** Diabetes mellitus, Self-care practice, Diabetes knowledge, Glycemic control, Ethiopia

## Abstract

**Objective:**

To assess the self-care practices and associated factors among diabetic patients in West Ethiopia.

**Results:**

A total of 252 study participants were included in the study, of this 54.8% were male. Of the participants more than half 150 (59.5%) had poor glycemic control and 153 (60.7%) of the participants had good self-care. Majority of the study participants 209 (82.9%) had adequate foot care and more than half 175 (69.4%) and 160 (63.5%) had adequate dietary plan and exercise management respectively. However of the total diabetic patients only 38 (15.1%) had adequate blood glucose testing practices. On multivariable logistic analysis poor self-care practices were more likely to occur among male patients (AOR = 5.551, 95% CI = 2.055–14.997, p = 0.001), patients living in rural area (AOR = 5.517, 95% CI = 2.184–13.938, p < 0.001), patients with duration of diabetes < 6 years (AOR = 41.023, 95% CI = 7.373–228.257, p < 0.001), patients with no access for self-monitoring blood glucose (AOR = 9.448, 95% CI = 2.198–40.617, p = 0.003), patients with poor knowledge about diabetes (AOR = 67.917, 95% CI = 8.212–561.686, p < 0.001) and patients with comorbidities (AOR = 18.621, 95% CI = 4.415–78.540, p < 0.001).

## Introduction

Diabetes care is complex and requires that many issues, beyond glycemic control, be addressed [1]. The American Diabetes Association’s (ADA’s) Standards of Care are intended to provide clinicians, patients, researchers, payers, and other interested individuals with the components of diabetes care, general treatment goals, and tools to evaluate the quality of care. The management plan should recognize diabetes self-management education (DSME) and ongoing diabetes support as integral components of care [1–3].

According to the 2017 International Diabetes Federation (IDF) update, by the end of 2017, 4 million deaths will happen as a result of diabetes and its complications. Alongside other non-communicable diseases, diabetes is increasing most markedly in the cities of low and middle income countries. The IDF South-East Asia and Western Pacific regions are at the epicentre of the diabetes crisis: China alone has 121 million people with diabetes and India’s diabetes population totals 74 million. African, Middle Eastern and Northern African and South-East Asian regions are expected to face the highest upsurge in the next 28 years. People from these regions develop disease earlier, get sicker and die sooner than their counterparts in wealthier nations [4, 5].

Previous studies had indicated that factors like older age, being male, lack of family/social support, lack of education, lack of knowledge about diabetes, presence of complications, being unemployed, poor adherence, lack of access for glucometer non-adherence to diet and exercise were significantly associated with poor self-care practices [6–17].

This study aimed at assessing the magnitude of self-care practices and factors affecting self-care practices among diabetic patients; hence such types of data reveals the magnitude of the problem and are important for the care delivery services so as to fill the gaps to resolve the problem.

## Main text

### Methods and materials

#### Study design and period

A facility based cross-sectional study was conducted from February 20 to May 20, 2016.

#### Study population

Adult diabetic patients who were on active follow up in DM clinic during the study period, Nekemte Referral Hospital.

#### Sample size determination and sampling technique

The required sample size was determined by considering the following assumptions for interview questionnaires: Sample size is calculated by taking the proportion of poor glycemic control which is 73.1% on diabetes patients at Diabetes Clinic of Jimma University Specialized Hospital (JUSH) [11]. With 95% confidence level and 5% margin of error to get an optimum sample size.

$${\text{n}} = \frac{{\left( {{\text{Z}} -\upalpha/2} \right)^{2} {\text{P}}\left( {1 - {\text{P}}} \right)}}{{{\text{d}}^{2} }} = \frac{{\left( {1.96} \right)^{2} 0.731\left( {1 - 0.731} \right)}}{{\left( {0.05} \right)^{2} }} = 302$$

Since the source population consisted of less than 10,000 respondents, the sample size was adjusted by using correction formula

$${\text{nf}} = {\text{n}}/1 + {\text{n}}/{\text{N}}$$

where nf = the final sample size, n = desired sample size 302 and N = total diabetic patients (941). The calculated sample size was nf = 229. Considering a 10% non-response rate, 252 diabetic patients were included in the study.

#### Inclusion and exclusion criteria

All diabetic adult patients of age greater than or equal to 15 years who attended chronic care department for diabetic care at least for 1 year were included while diabetic patients who were critically ill, psychotic and/or unable to communicate with data collector due to other underlying medical disorder were excluded.

#### Data collection tool

To collect primary data, questionnaires and interview was used in the study. Data collection format was developed by the principal investigator to collect the blood glucose measurements and anti-diabetic medications used by respective study subjects. The questionnaire was developed after literatures were reviewed thoroughly [10–16].

#### Definitions of terms

##### Self-care practices

Self-care practices refer to behaviors such as following a diet plan, increased exercise, self-blood glucose testing, and foot care [18]. Details about self-care activities were collected using the Summary Diabetes Self-Care Activities (SDSCA) [19] questionnaire after minor changes were made to it to suit the Ethiopian context. Four domains (diet, exercise, foot care and blood glucose testing) of self-care practices were used to assess the self-care practices of diabetic patients to diabetes. For all domains frequency of self-care activity in the last 7 days were measured. For each domain the mean was calculated and categorized as adequate for scores above mean value and unsatisfactory for scores less than mean value and presented as tables in result. Accordingly after calculating mean score, patients had adequate diet plan if scored ≥ 4, patients had adequate foot care if scored ≥ 7, patients had adequate exercise if scored ≥ 4, patients had adequate self-blood glucose testing if scored ≥ 1. The overall mean score was calculated by summation of the mean score for diet, exercise, foot care and blood glucose testing divided by the sum of number of questions under each scale. After calculating an overall mean score, it was classified as having good self-care practice if the patient scored ≥ 4 or poor self-care practice if the patient scored < 4.

##### Glycemic control

Glycemic control was assessed by using Fasting Blood Glucose (FBG) level. The glycemic recommendation for non-pregnant adults is in between 70 and 130 mg/dl, when the patients FBG was beyond this value we considered as poor glycemic control according to ADA [3].

##### Diabetes knowledge

The Diabetes Knowledge Test (DKT) was utilized to assess diabetic patients’ general understanding of their disease and treatment recommendations. The DKT was developed and tested for reliability and validity by the University of Michigan scholars and was adapted for the Ethiopian context. DKT consisting of 23 questions has been shown to adequately estimate general patient knowledge of diabetes. Only the first 14 questions were applied to patients who do not use insulin and the entire questionnaire were administered to patients who use insulin. The score for each participant was determined by dividing the number of correct answers by the total number of questions (14 for those receiving only oral hypoglycemic agents and 23 questions for patients taking insulin). To assess the level of knowledge of diabetes, we recorded the patients’ level of knowledge into three groups on the basis of their DKT scores: as good, acceptable and poor knowledge if their overall score is ≥ 75%, 60–74%, and ≤ 59%, respectively. The scores were used to determine overall knowledge level [20].

##### Body mass index (BMI)

Body mass index (BMI) was categorized as normal weight if BMI was 18.5–24.9, Underweight if BMI was < 18.5, overweight if BMI was 25–29.9 kg/m^2^, and obese if BMI was ≥ 30 kg/m^2^ based on the World Health Organization criteria [21].

### Results

A total of 252 study participants were included in the study, of this 54.8% were male. The mean age of the participants was 41.7 ± 17.6 years. Out of the study participants 52.0% were in the age range of 30–60 years. More than half of the study participants were unemployed 131 (52.0%) and 129 (51.2%) were from urban (Table 1).

**Table 1:** Socio-demographic and clinical characteristics of diabetic patients on follow up at Nekemte Referral Hospital, West Ethiopia, from February 20 to May 20, 2016 (n = 252)

Variables	Category	Frequency	Percentage
Sex	Male	138	54.8
	Female	114	45.2
Age	< 30	89	35.3
	30–60	131	52.0
	> 60	32	12.7
Educational status	No formal education	70	27.8
	Primary school	97	38.5
	Secondary school	48	19.0
	College/University	37	14.7
Occupation	Employed	48	19.0
	Unemployed	131	52.0
	Farmer	73	29.0
Residence	Urban	129	51.2
	Rural	123	48.8
BMI	< 18.5 (Underweight)	17	6.7
	18.5–24.9 (Normal weight)	142	56.3
	25–29.9 (Overweight)	50	19.8
	≥ 30 (Obese)	43	17.1
Family/social support	Yes	64	25.4
	No	188	74.6
Family history of diabetes	Yes	32	12.7
	No	220	87.3
Duration of diabetes	< 6	154	61.1
	6–10	69	27.4
	> 10	29	11.5
Number of medications taken	1	138	54.8
	≥ 2	114	45.2
Access for self-monitoring blood glucose	Yes	26	10.3
	No	226	89.7
Hospitalization due to diabetic related problem	Yes	53	21.0
	No	199	79.0
Knowledge of diabetes	Good	30	11.9
	Acceptable	53	21.0
	Poor	169	67.1
Anti-diabetic medication	Metformin	57	22.6
	Insulin	159	63.1
	Insulin and metformin	10	4.0
	Metformin and Glibenclamide	21	8.3
	Glibenclamide	5	2.0
Presence of comorbidities	Yes	75	30.6
	No	175	69.4
Type of diabetes mellitus	Type 1	159	63.1
	Type 2	93	36.9
Glycemic control	≤ 130	102	40.5
	> 130	150	59.5
Self-care	Poor self-care	99	39.3
	Good self-care	153	60.7

Majority of the study participants 188 (74.6%) had no family/social support and most of them 220 (87.3%) had no family history of diabetes. More than half 61.1% of the participants treated for diabetes mellitus for less than 6 years. Only 10.3% of the participants had access for monitoring their blood glucose. About 159 (63.1%) of the patients were taking only insulin. Majority 169 (67.1%) of the participants had poor knowledge about diabetes. Of the participants 159 (63.1%) patients had type 1 DM. More than half of the participants 150 (59.5%) had poor glycemic control. One hundred fifty-three (60.7%) participants had good self-care (Table 1).

Regarding self-care practice domains of diabetic patients majority of them 209 (82.9%) had adequate foot care and more than half 175 (69.4%) and 160 (63.5%) had adequate dietary plan and exercise management respectively. However of the total diabetic patients only 38 (15.1%) had adequate blood glucose testing practices (Table 2).

**Table 2:** Distribution of self-care practice domains diabetic patients on follow up at Nekemte Referral Hospital, West Ethiopia, from February 20 to May 20, 2016 (n = 252)

Self-care practice domains	Adequate	Unsatisfactory
Diet	175 (69.4%)	77 (30.6%)
Exercise	160 (63.5%)	92 (36.5%)
Foot care	209 (82.9%)	43 (17.1%)
Blood glucose testing	38 (15.1%)	214 (84.9%)

Variables like sex, educational status, occupation, residence, BMI, knowledge of diabetes and type of DM had shown statistically significant association (p < 0.05) with self-care practices in bivariable analysis. In this study, variables with p-value < 0.25 were entered into multivariable analysis to identify independent predictors of poor self-care practices among diabetic patients (Table 3).

**Table 3:** Logistic regression analysis of factors associated with self-care practices among diabetic patients on follow up at Nekemte Referral Hospital, West Ethiopia, from February 20 to May 20, 2016 (n = 252)

Variables	Categories	Self-care practice		COR (95% CI) p value	AOR (95% CI) p value
		Good	Poor		
Sex	Male	76	62	1.698 (1.013–2.844) p = 0.044	5.551 (2.055–14.997) p = 0.001
	Female	77	37	1.00	1.00
Age	< 30	48	41	0.854 (0.380–1.917) p = 0.702	–
	30–60	89	42	0.472 (0.215–1.034) p = 0.060	–
	> 60	16	16	1.00	–
Educational status	No formal education	36	34	1.744 (0.767–3.966) p = 0.185	1.826 (0.334–9.988) p = 0.487
	Primary school	51	46	1.665 (0.760–3.646) p = 0.202	2.026 (0.471–8.718) p = 0.343
	Secondary school	32	16	0.264 (0.089–0.784) p = 0.037	0.127 (0.018–1.098) p = 0.063
	College/University	24	13	1.00	1.00
Occupation	Employed	38	10	1.00	–
	Unemployed	84	47	2.126 (0.972–4.651) p = 0.059	–
	Farmer	31	42	5.148 (2.229–11.890) p < 0.001	–
Residence	Urban	99	30	1.00	1.00
	Rural	54	69	4.217 (2.453–7.250) p < 0.001	5.517 (2.184–13.938) p < 0.001
BMI	< 18.5	15	2	1.00	–
	18.5–24.9	70	72	7.714 (1.701–34.978) p = 0.008	–
	25–29.9	34	16	3.529 (0.719–17.317) p = 0.120	–
	≥ 30	34	9	1.985 (0.382–10.319) p = 0.415	–
Family/social support	Yes	33	31	1.00	–
	No	120	68	0.603 (0.340–1.070) p = 0.084	–
Family history of diabetes	Yes	15	17	1.00	–
	No	138	82	0.524 (0.249–1.106) p = 0.090	–
Duration of diabetes	< 6	56	98	2.154 (0.966–4.804) p = 0.061	41.023 (7.373–228.26) p < 0.001
	6–10	27	42	1.125 (0.627–2.018) p = 0.693	2.768 (0.966–7.933) p = 0.058
	> 10	16	13	1.00	1.00
Number of medications taken	1	78	60	1.00	–
	≥ 2	75	39	0.676 (0.405–1.129) p = 0.135	–
Access for self-monitoring BG	Yes	19	7	1.00	1.00
	No	134	92	1.864 (0.753–4.613) p = 0.178	9.448 (2.198–40.617) p = 0.003
Hospitalization due to diabetic related problem	Yes	30	23	1.241 (0.672–2.292) p = 0.491	–
	No	123	76	1.00	–
Knowledge of diabetes	Good	28	2	1.00	1.00
	Acceptable	41	12	4.098 (0.851–19.738) p = 0.079	0.903 (0.104–7.864) p = 0.926
	Poor	84	85	14.167 (3.271–61.36) p < 0.001	67.917 (8.212–561.686) p < 0.001
Anti-diabetic medication	Metformin	35	22	1.00	–
	Insulin	85	74	1.385 (0.747–2.569) p = 0.301	–
	Insulin and metformin	8	2	0.398 (0.077–2.048) p = 0.270	–
	Metformin and glibenclamide	21	0	0.000	–
	Glibenclamide	4	1	0.398 (0.042–3.793) p = 0.423	–
Presence of comorbidities	Yes	42	35	1.445 (0.839–2.490) p = 0.184	18.621 (4.415–78.540) p < 0.001
	No	111	64	1.00	1.00
Type of DM	Type 1	85	74	2.368 (1.360–4.122) p = 0.002	–
	Type 2	68	25	1.00	–
Glycemic control	≤ 130	64	38	1.00	–
	> 130	89	61	1.154 (0.688–1.936) p = 0.586	–

According to the result of multivariable logistic analysis poor self-care practices were more likely to occur among male patients (AOR = 5.551, 95% CI = 2.055–14.997, p = 0.001), patients living in rural area (AOR = 5.517, 95% CI = 2.184–13.938, p < 0.001), patients with duration of diabetes < 6 years (AOR = 41.023, 95% CI = 7.373–228.257, p < 0.001), patients with no access for self-monitoring blood glucose (AOR = 9.448, 95% CI = 2.198–40.617, p = 0.003), patients with poor knowledge about diabetes (AOR = 67.917, 95% CI = 8.212–561.686, p < 0.001) and patients with comorbidities (AOR = 18.621, 95% CI = 4.415–78.540, p < 0.001) (Table 3).

### Discussion

Self-monitoring of glycemic control is a cornerstone of diabetes care that can ensure patient participation in achieving and maintaining specific glycemic targets. Self-monitoring provides information about current glycemic status, allowing for assessment of therapy and guiding adjustments in diet, exercise and medication in order to achieve optimal glycemic control [22].

This study revealed that the self-care practices of diabetic patients accounts for 60.7%. Self-care practices in this study is higher as compared to previous done studies in Harari [17], JUSH [23], India [24], which reported 50.9%, 39.2%, 46.4% of self-care practices; but lower than study done in Nigeria [25] which reported 79.5% of self-care practices. The differences in self-care practices could be due to easier access to health-related activities and higher proportions of literate population in the present study setting as compared to previously done studies in Ethiopia.

This study showed that male patients were 5.551 times more likely to had poor self-care practices as compared to female patients. This finding is consistent with that reported by other studies from Tikur Anbesa Specialized hospital [16], Nigeria [25] and Bangladesh [26]. This difference in gender shows difference in awareness over self-care practices and commitment for adhering to the self-care practices, thus education on self-care practices has to be provided for all diabetic patients.

The living places of the patients had shown significance association with poor self-care practices. Patients living in rural areas are 5.517 times more likely to develop poor self-care practices than those living in urban. Similar findings were reported from Ayder Comprehensive Specialized Hospital [27] and Bangladesh [26].

Patients with shorter duration of diabetes had shown significant association with poor self-care practices. Similar findings were reported from Ayder Comprehensive Specialized Hospital [27], Bangladesh [26] and United Arab Emirates [28]. The reasons for poor self-care in patients with shorter duration of diabetes could be due to less regular counseling and contact with health professionals that may help them to create their awareness for self-care practices.

Patients with no access for self-monitoring blood glucose were 9.448 times more likely to had poor self-care practices than those who had access for self-monitoring blood glucose. This finding is consistent with studies done at JUSH [23], California [29] and United Kingdom [30]. The reason for no access of self-monitoring blood glucose could be because of low socioeconomic status of the study participants and lack of their awareness on the use of glucometer.

Poor knowledge about diabetes was associated significantly with poor self-care practices. This finding is consistent with other studies done in Tikur Anbesa Specialized hospital [16] and Bangladesh [31]. This could be explained as patients with poor knowledge about diabetes are less compliant to their medication and self-care practice and this will result in poor glycemic control [10, 32, 33].

### Conclusion

The present study concluded that self-care practices of study participants were poor. In particular blood glucose testing domain of self-care practice was very poor and relatively there were good foot care among the study participants. Thus we recommend that health care providers should begin by taking time to evaluate their patients’ perceptions and make realistic and specific recommendations for self-care activities.

## Limitations

Data about diabetes and self-care knowledge were self-reported; this method has the disadvantages of recall bias and eliciting only socially acceptable responses and hence, may, lead to overestimation of some of the results. The study period might be short but all the diabetic patients who came to hospital within study period and satisfied the inclusion criteria.

## Abbreviations

AOR: adjusted odds ratio
BMI: body mass index
COR: crude odds ratio
DKT: Diabetes Knowledge Test
DM: diabetes mellitus
FBG: fasting blood glucose
HbA1c: glycosylated hemoglobin
MCQ: multiple choice questions
NRH: Nekemte Referral Hospital
SDSCA: Summary Diabetes Self-Care Activities
SPSS: Statistical Program for the Social Sciences
WHO: World Health Organization
